# Optical multistability and Fano line-shape control via mode coupling in whispering-gallery-mode microresonator optomechanics

**DOI:** 10.1038/srep39781

**Published:** 2017-01-03

**Authors:** Suzhen Zhang, Jiahua Li, Rong Yu, Wei Wang, Ying Wu

**Affiliations:** 1 School of Physics, Huazhong University of Science and Technology, Wuhan 430074, People’s Republic of China; 2 College of physics and Electronic Information, Dezhou University, Dezhou 253023, People’s Republic of China; 3 MOE Key Laboratory of Fundamental Physical Quantities Measurement, Huazhong University of Science and Technology, Wuhan 430074, People’s Republic of China; 4 School of Science, Hubei Province Key Laboratory of Intelligent Robot, Wuhan Institute of Technology, Wuhan 430073, People’s Republic of China; 5 Shanghai Institute of Optics and Fine Mechanics, Chinese Academy of Sciences, Shanghai 201800, People’s Republic of China

## Abstract

We study a three-mode (i.e., a clockwise mode, a counterclockwise mode, and a mechanical mode) coherent coupling regime of the optical whispering-gallery-mode (WGM) microresonator optomechanical system by considering a pair of counterpropagating modes in a general case. The WGM microresonator is coherently driven by a strong control laser field and a relatively weak probe laser field via a tapered fiber. The system parameters utilized to explore this process correspond to experimentally demonstrated values in the WGM microresonator optomechanical systems. By properly adjusting the coupling rate of these two counterpropagating modes in the WGM microresonator, the steady-state displacement behaviors of the mechanical oscillation and the normalized power transmission and reflection spectra of the output fields are analyzed in detail. It is found that the mode coupling plays a crucial role in rich line-shape structures. Some interesting phenomena of the system, including optical multistability and sharp asymmetric Fano-shape optomechanically induced transparency (OMIT), can be generated with a large degree of control and tunability. Our obtained results in this study can be used for designing efficient all-optical switching and high-sensitivity sensor.

Cavity optomechanical system, a rapidly developing regime of research which concerns the coherent coupling between optical modes and mechanical modes via the radiation pressure of photons trapped in an optical cavity (for recent reviews, see, e.g., refs 1, 2, 3, 4, 5), has made the rapid advance of technology in recent years. The marked achievements have been made in those optical components, such as ultrahigh-precision measurement^6^, gravitation-wave detection^7^, quantum information processing (QIP)^8^,^9^,^10^, higher-order sidebands^11^,^12^,^13^, optical nonlinearity^14^,^15^,^16^, mechanical parity-time 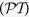 symmetry^17^,^18^,^19^ and 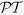-broken chaos^20^, quantum entanglement^21^,^22^,^23^,^24^,^25^,^26^, optomechanically induced transparency (OMIT)^27^,^28^,^29^,^30^,^31^,^32^,^33^,^34^,^35^, and optomechanically induced stochastic resonance (OMISR)^36^, and many others^1^,^2^,^3^,^4^,^5^.

On the other hand, there is also an increasing interest in the nonlinear optical phenomena based on quantum coherence and interference in optomechanics. One of the most interesting phenomena, optical bistability and multistability^37^,^38^,^39^,^40^, have attracted a lot of attention due to a variety of fundamental studies and practical applications in optical communication and optical computing, such as all-optical switching^41^,^42^, sensitive force or displacement detections and memory storage^43^,^44^, etc. Another one of the curious optical phenomena is based on quantum coherence and interference, i.e., the so-called Fano resonance^45^, the pronounced feature of which is a sharp asymmetric line profile. It was first theoretically explained by Ugo Fano^46^. He discovered that the shape of this resonance, which is based on the interaction of a discrete excited state of an atom with a continuum of scattering states, is quite different from the resonance which generally described by the Lorentzian formula. Because of the asymmetry of the Fano line-shape and the enhanced interference effect, many theoretical^47^,^48^,^49^,^50^,^51^,^52^,^53^ and experimental^54^,^55^,^56^,^57^,^58^,^59^ works have be done so far.

The rapid progress of the nonlinear optics and the optomechanical system is mainly due to the development of various optical microcavities^2^,^60^,^61^,^62^, which have the small mode volume, small masses, high quality *Q* factor, and high on-chip integrability. It is wroth pointing out that, different from the standing wave in a Fabry-Pérot cavity, optical whispering-gallery-mode (WGM) microresonator supports a pair of counterpropagating modes: clockwise (CW) mode and counterclockwise (CCW) mode^63^,^64^, which have a degenerate frequency and an identical mode field distribution. As a result, the propagating direction of the light in the coupling region determines which mode is coherently excited. Due to residual scattering of light at the surface or in the bulk glass, the counterpropagating mode can also be significantly populated. In the past, the above-mentioned OMIT effect in a WGM toroidal microcavity has been widely studied based on the coupling of a single stationary mode in the normal mode basis with a mechanical mode^27^ where the two modes are well resolved under the condition that 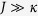 (namely, in the ultrastrong coupling regime, here *J* is the coupling rate between the two CW and CCW modes and *κ* is the total cavity decay rate, respectively). In this limit, only one of the two stationary modes is considered and the other is neglected since the neglected mode is far-off-resonant and therefore it is not populated^27^. Correspondingly, the experimental conditions also have to be chosen in order to avoid a mechanical sideband coinciding with the resonance frequency of the neglected mode.

Whereas little has been discussed about and done with the three-mode (a CW mode, a CCW mode, and a mechanical mode) coherent coupling regime of the WGM microresonator optomechanical system by considering a pair of counterpropagating modes in a more general case. Specifically, the studied circumstances involves two aspects as follows. (i) The two modes cannot be resolved and hence two of them are populated when *J* ≤ *κ* (in the weak coupling regime); (ii) We also consider the condition of *J* > *κ* (the strong coupling regime) but not the ultrastrong coupling regime 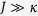 (i.e., the transition coupling region *κ* < *J* < 3*κ*), thus the two modes still can be resolved. In view of these factors, the main aim of the present work is to take into account the simultaneous coupling of the two modes with a mechanical oscillation for the cases of *J* ≤ *κ* and *J* > *κ*, which relaxes the mode coupling rate required to reach 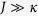 27 and makes the proposed device suitable for practical applications. We present a complete analytical investigation based on a three-mode WGM microresonator optomechanics and on a perturbation approach to calculate the power transmission and reflection spectra of a weak probe field under the action of a strong control field. We mainly study the mode-coupling effect on the properties of the mechanical oscillator displacement, the light forward transmission, and backward reflection. We clearly show that the mode coupling characterized by *J* can lead to some interesting phenomena of the on-chip WGM microresonator optomechanical system, such as optical multistability and sharp asymmetrical Fano-shape OMIT resonances. We also discuss the influences of the other system parameters including the power of the control field, the outgoing coupling coefficient, and the optomechanical coupling strength on all-optical generation of Fano line-shapes. Our findings provide new insights into the aspects of the interaction between the CW or CCW light and mechanical motion. It can be used to design low-power all-optical switches, modulators, and high-sensitivity sensors.

## Results

### Theoretical model

**Figure 1:**
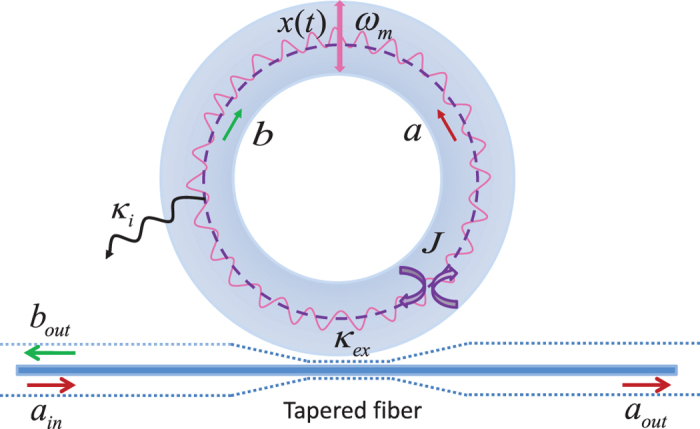
Schematic diagram of the WGM microresonator optomechanical system consisting of a tapered fiber and a WGM ring microresonator which contains a mechanical breathing mode with resonance frequency Ω_*m*_. Two degenerate counterpropagating modes are respectively labeled as 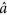 (CCW) and 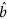 (CW) with the same frequency *ω*. Because of internal defect centers or surface roughness, these two modes are coupled to each other at a rate *J*, which is known as the so-called mode coupling. The intrinsic loss of the cavity fields is denoted by *κ*_*i*_ and the waveguide-cavity coupling strength is *κ*_*ex*_. The CCW mode is driven by an external input field *a*_*in*_ including a strong control field and a weak probe field with field strengths *ε*_*c*_ and *ε*_*p*_ as well as carrier frequencies *ω*_*c*_ and *ω*_*p*_. The output fields are described by *a*_*out*_ and *b*_*out*_, respectively. See text for details.

As schematically shown in Fig. 1, we consider a microresonator optomechanical system, which consists of a WGM microresonator containing a mechanical breathing mode and a tapered fiber. As shown in ref. 27, the WGM microresonator can support counterclockwise (CCW) and clockwise (CW) propagating modes, which are described in terms of the annihilation (creation) operators 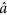 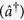 and 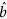 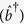 with a common frequency *ω*.

Because of residual scattering of light at the surface or in the bulk glass, the two CCW and CW propagating modes are coupled to each other at a rate *J*. At the same time, these two modes interact with the mechanical radial breathing mode through the radiation pressure, where the optomechanical coupling strength between the optical modes and the mechanical mode is characterized by *G*. The two CCW and CW modes are side-coupled to a tapered fiber by the evanescent field which is determined by the propagating direction of the light in the coupling region. We assume that the CCW mode in the WGM microresonator (see Fig. 1) is coherently driven by an external input laser field consisting of a strong control field and a relatively weak probe field, denoted by 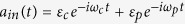 with the field strengths (carrier frequencies) *ε*_*c*_ and *ε*_*p*_ (*ω*_*c*_ and *ω*_*p*_). The field strengths *ε*_*c*_ and *ε*_*p*_ are normalized to a photon flux at the input of the microresonator and are defined as 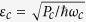 and 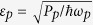, where *P*_*c*_ and *P*_*p*_ are the powers of the control field and the probe field, respectively. Without loss of generality, we assume that *ε*_*p*_ and *ε*_*c*_ are real. Experimentally, *ε*_*p*_ is usually chosen to be much smaller than *ε*_*c*_. More information on the device and experimental details can be found in ref. 27 and supporting online material accompanying ref. 27. The Hamiltonian of the whole system is given by


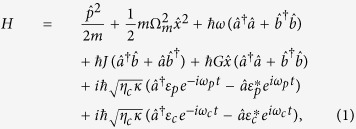


where 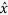 and 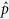 are the position and momentum operators of the mechanical oscillator with the effective mass *m* and resonance frequency Ω_*m*_, satisfying the relationship 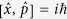. The optomechanical coupling constant *G* between the mechanical and cavity modes can be defined as *G* = −∂*ω*/∂*x*, which is determined by the shift of the cavity resonance frequency per the displacement of the mechanical resonator^2^. The total decay rate of the WGM microresonator mode (the microresonator linewidth) is denoted by *κ* = *κ*_*i*_ + *κ*_*ex*_, where *κ*_*i*_ is the intrinsic decay rate, related to the intrinsic quality factors *Q*_*i*_ by *κ*_*i*_ = *ω/Q*_*i*_ and *κ*_*ex*_ is the external decay rate (the outgoing coupling coefficient) from the optical resonator into the tapered fiber, related to the coupling quality factor *Q*_*e*_ by *κ*_*e*_ = *ω/Q*_*e*_. The total decay rate *κ* is related to the total quality factor *Q* by *κ* = *ω/Q*. Obviously, 1/*Q* = 1/*Q*_*i*_ + 1/*Q*_*e*_. Various techniques have been reported for changing Q dynamically^65^,^66^,^67^. The outgoing coupling coefficient *η*_*c*_ = *κ*_*ex*_/*κ* can be used to measure the cavity loading degree. (i) If *η*_*c*_ < 0.5, the WGM microresonator is in the under-coupling regime. (ii) If *η*_*c*_ = 0.5, the WGM microresonator is in the critical-coupling regime. (iii) If *η*_*c*_ > 0.5, the WGM microresonator is in the over-coupling regime. The outgoing coupling coefficient *η*_*c*_ can be continuously tuned by changing the air gap between the WGM microresonator and tapered fiber^27^. Finally, it should be pointed out that the coupling between the CW and CCW modes is usually caused by residual scattering of light at the surface or in the bulk glass as well as the case when there are interruptions (such as nanoparticles). Thus the surface roughness or internal defect center in the WGM microresonator is the critical point to make the coupling between the CCW and CW modes. These factors above may be used to control and tune the coupling rate *J*. Note that both *J* and *κ* can be controlled independently in actual systems.

In the above Hamiltonian (1), the first and second terms represent the energies of the mechanical oscillator. The third term is the energy of the WGM microresonator. The fourth term describes the coherent coupling of the CCW mode 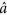 with the CW mode 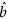, i.e., the so-called mode coupling term. The fifth term presents the optomechanical coupling due to the radiation pressure with the coupling strength *G*. The last two terms in Eq. (1) describe the interactions between the cavity field and the two input fields, respectively.

### Controlled optical bistability and multistability in WGM microresonator optomechanical system

In this section, as the first insight, we will show how optical bistability and multistability for the displacement of the mechanical resonator can be modified and controlled by the mode coupling rate *J* under the action of the strong control field in our proposed scheme.

When the coupled system is strongly driven, it can be characterized by the semiclassical steady-state solutions with large amplitudes for both optical and mechanical modes. In view of this, by calculating Eqs (8, 9, 10) of the Methods under steady-state conditions, we have the result for 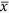



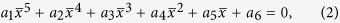


with


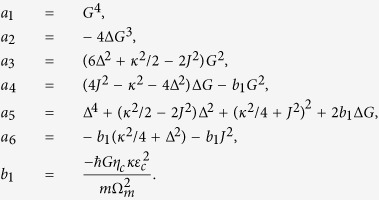


It can be clearly see from Eq. (2) that the steady-state value 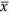 for the displacement of the mechanical resonator is a fifth-order polynomial equation, thus, there are at most five real roots, significantly different from the previous studies (a cubic equation that can have up to three real roots)^11^,^39^,^40^,^41^. The reason for this is that the two CW and CCW modes simultaneously coupled with the mechanical oscillator.

**Figure 2:**
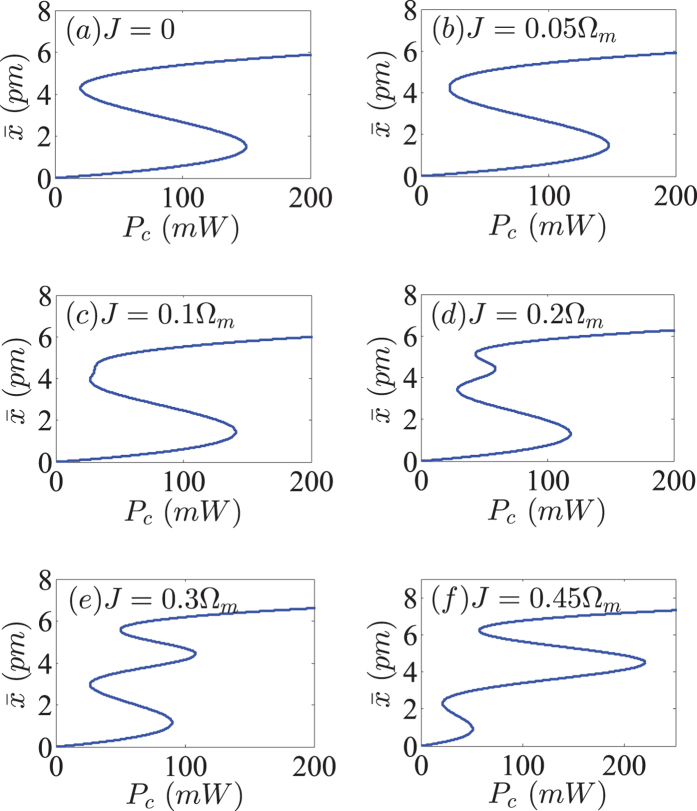
The displacement of the mechanical resonator 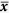 as a function of the power of the control field *P*_*c*_ for the six different values of the mode coupling rate *J*. For the numerical simulations, we use the following experimental parameters from ref. 27: *m* = 20 ng, *G*/2*π* = −12 GHz/nm, Γ_*m*_/2*π* = 41 kHz, *κ*/2*π* = 15 MHz, Ω_*m*_/2*π* = 51.8 MHz, *η*_*c*_ = 0.5, and Δ = −Ω_*m*_, respectively. The wavelength of the control field is chosen to be *λ*_*c*_ = 532 nm.

We plot the stationary value for the displacement of the mechanical resonator 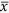 as a function of the power of the control field *P*_*c*_ for the six different values of the coupling rate *J* between the two CW and CCW modes as shown in Fig. 2(a–f). First of all, from Fig. 2(a) corresponding to the case of *J* = 0, it is easy to see that an S-shaped behavior of the displacement of the mechanical resonator can be formed efficiently, that is to say, the coupled system exhibits the bistable behavior where the largest and smallest roots of 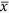 are stable, and the middle one is unstable. Note that, such an optical bistability has been investigated in the previous optomechanical systems^11^,^39^,^40^,^41^. In this situation, the system only has a single bistable window. This is because only one CCW mode of the GWM microresonator is coupled with the mechanical resonator while the other CW mode coupled with the mechanical resonator is usually neglected^27^. When considering *J* = 0.05 Ω_*m*_ in Fig. 2(b), it is expected that optical bistable behavior is not changed almost, due to a significantly small increased value of the mode coupling rate *J*. Second, as we continue to increase the coupling rate *J* between these two CW and CCW modes, e.g., *J* = 0.1 Ω_*m*_ and 0.2 Ω_*m*_, optical multistable behavior begins to appear, as can be seen in Fig. 2(c) and (d). For the case that *J* = 0.3 Ω_*m*_ in Fig. 2(e), the coupled system obviously displays optical multistabile behavior. Lastly, with further increasing *J* (for example, *J* = 0.45 Ω_*m*_), the system also exhibits the multistability consisting of the two separated bistable windows as depicted in Fig. 2(f). It is shown from Fig. 2(a–f) that the steady-state response of the mechanical resonator may be bistable and tristable, depending strongly on the value of the mode coupling rate *J*. Here it is worth pointing out that in the region with three solutions, two of them are stable by a standard linear stability analysis^68^. While in the region with five solutions, three of them are stable. As a consequence, these results for 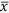 represent bistable [see Fig. 2(a,b)] and tristable [see Fig. 2(d–f)] regimes, respectively. Finally, for the threshold values of *J* between the bistable and triplestable regimes, they are too cumbersome to be given here.

According to what has been discussed above, we can arrive at the conclusion that the generated bistability and tristability in Fig. 2 are closely related to the mode coupling rate *J*. The WGM microresonator optomechanics we consider here enables more controllability in the bistable and tristable behaviors of the displacement of the mechanical resonator by appropriately adjusting the coupling rate *J* between the two modes of the GWM microresonator. From an experimental point of view, the controlled triple-state switching is possible practically by adding a pulse sequence onto the input field^69^. Such an optical tristability can be used for building all-optical switches, logic-gate devices, and memory devices for optical computing and quantum information processing.

### Controlled sharp asymmetric Fano resonance OMIT line-shapes in WGM microresonator optomechanical system

**Figure 3:**
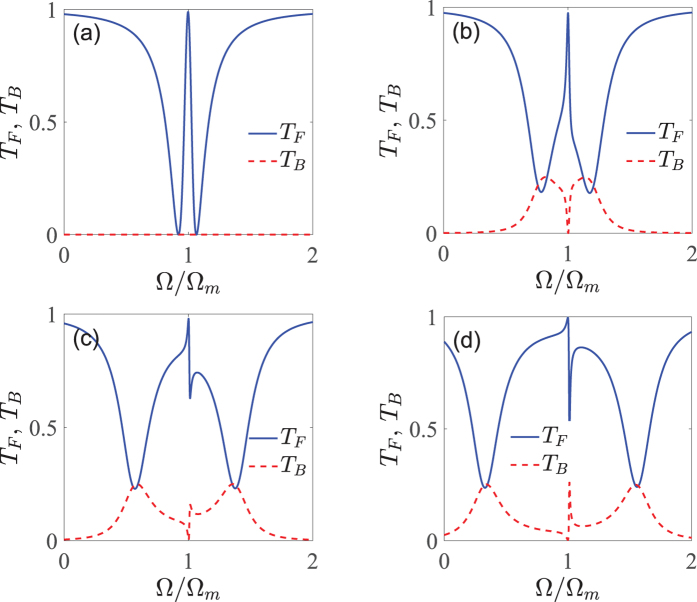
The normalized power forward transmission *T*_*F*_ (the blue line) and backward reflection *T*_*B*_ (the red dot line) as a function of the detuning Ω for the four different values of the mode coupling rate *J*. (**a**) *J* = 0, (**b**) *J* = 0.2 Ω_*m*_, (**c**) *J* = 0.4 Ω_*m*_, and (**d**) *J* = 0.6 Ω_*m*_. In all the figures, we employ *P*_*c*_ = 10 mW, *m* = 20 ng, *G*/2*π* = −12 GHz/nm, Γ_*m*_/2*π* = 41 kHz, *κ*/2*π* = 15 MHz, Ω_*m*_/2*π* = 51.8 MHz, *η*_*c*_ = 0.5, and Δ = −Ω_*m*_, respectively.

**Figure 4:**
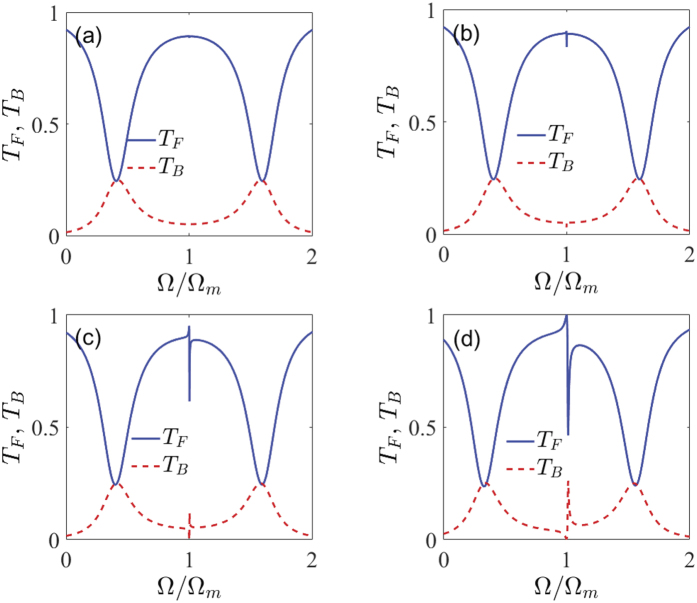
The normalized power forward transmission *T*_*F*_ (the blue line) and backward reflection *T*_*B*_ (the red dot line) as a function of the detuning Ω for the four different values of the power of the control field *P*_*c*_. (**a**) *P*_*c*_ = 10 *μ*W, (**b**) *P*_*c*_ = 100 *μ*W, (**c**) *P*_*c*_ = 1 mW, and (**d**) *P*_*c*_ = 10 mW. In all the figures, we employ *J* = 0.6 Ω_*m*_, *m* = 20 ng, *G*/2*π* = −12 GHz/nm, Γ_*m*_/2*π* = 41 kHz, *κ*/2*π* = 15 MHz, Ω_*m*_/2*π* = 51.8 MHz, *η*_*c*_ = 0.5, and Δ = −Ω_*m*_, respectively.

**Figure 5:**
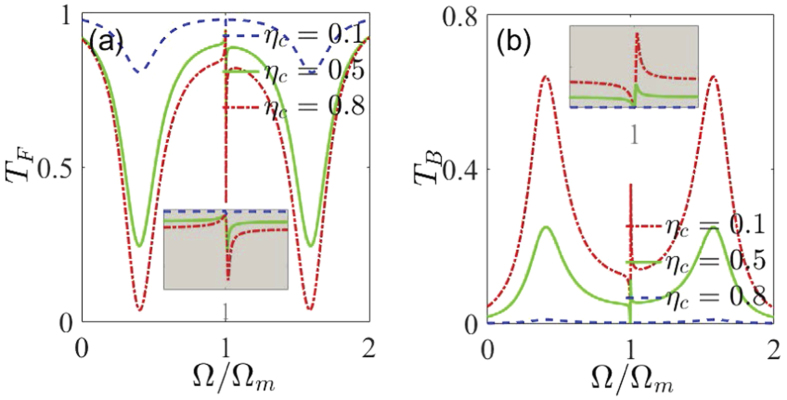
The normalized power (**a**) forward transmission *T*_*F*_ and (**b**) backward reflection *T*_*B*_ as a function of the detuning Ω for the three different values of the outgoing coupling coefficient *η*_*c*_: (i) *η*_*c*_ = 0.1 (under coupling), (ii) *η*_*c*_ = 0.5 (critical coupling), and (iii) *η*_*c*_ = 0.8 (over coupling). The insets in (**a**) and (**b**) show a magnified view of Fano line-shapes near Ω = Ω_*m*_ in a smaller region. We use *P*_*c*_ = 900 *μ*W, *J* = 0.6 Ω_*m*_, *m* = 20 ng, *G*/2*π* = −12 GHz/nm, Γ_*m*_/2*π* = 41 kHz, *κ*/2*π* = 15 MHz, Ω_*m*_/2*π* = 51.8 MHz, and Δ = −Ω_*m*_, respectively.

**Figure 6:**
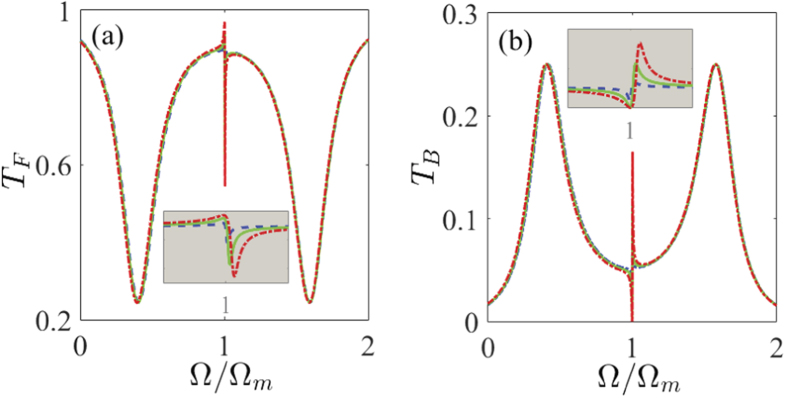
The normalized power (**a**) forward transmission *T*_*F*_ and (**b**) backward reflection *T*_*B*_ as a function of the detuning Ω for the three different values of the optomechanical coupling strength *G*. Here the blue double dots line is *G* = −6 GHz/nm, the green line is *G* = −12 GHz/nm, and the red dot dash line is *G* = −18 GHz/nm, respectively. The insets in (**a**) and (**b**) show a zoom-in plot of Fano line-shapes near Ω = Ω_*m*_ in a smaller region. We use *P*_*c*_ = 900 *μ*W, *J* = 0.6 Ω_*m*_, *m* = 20 ng, Γ_*m*_/2*π* = 41 kHz, *κ*/2*π* = 15 MHz, Ω_*m*_/2*π* = 51.8 MHz, *η*_*c*_ = 0.5, and Δ = −Ω_*m*_, respectively.

The Fano resonance, which has a pronounced sharp asymmetric line-shape profile, is remarkably different from the above-mentioned symmetric OMIT spectral profile^11^,^27^. Due to its sharp asymmetric line-shape, any small changes in the considered Fano system are able to cause the huge change of both the amplitude and phase. Consequently, the possibility of controlling and tuning the Fano resonance is a functionality of key relevance. In this section, we look at the effect of various system parameters on the asymmetric Fano resonance line-shapes of the normalized power forward transmission *T*_*F*_ and backward reflection *T*_*B*_ at the output of the device. The detailed results are given in Figs 3, 4, 5 and 6.

First of all, we start by exploring how the line-shapes of the Fano resonance can be modified by varying the coupling rate *J* between the two CW and CCW counterpropagating modes. Figure 3 shows the normalized power forward transmission *T*_*F*_ and backward reflection *T*_*B*_ as a function of the detuning Ω (Ω ≡ *ω*_*p*_ − *ω*_*c*_, in units of Ω_*m*_) under the four different values of the mode coupling rates *J* based on the obtained analytical expressions (27) and (28) of the Methods. We use the following parameter values *ε*_*p*_/*ε*_*c*_ = 0.05, *P*_*c*_ = 10 mW, *η*_*c*_ = 0.5, and the other system parameters are exactly the same as those in Fig. 2. Figure 3(a) shows the normalized power forward transmission *T*_*F*_ and backward reflection *T*_*B*_ versus the detuning Ω for the case of *J* = 0, which means that the two counterpropagating modes are not coupled to each other. It can be seen from Fig. 3(a) that the normalized power forward transmission *T*_*F*_ (see the blue line) has a single transparent peak in the center of Ω = Ω_*m*_ and two dips on both sides, which exhibits a symmetric dip-peak-dip spectral structure in the forward transmission. This phenomenon shows an obvious OMIT effect, which has been intensively studied in the previous works^27^,^28^,^29^,^30^,^31^,^32^,^33^,^34^,^35^. In the meantime, the normalized power backward reflection *T*_*B*_ (see the red dot line) is zero, as predicted. This is because the CW mode *b* (see Fig. 1) is not introduced at all when *J* = 0. It also corresponds to the same situation as that in Fig. 2(a) where the optomechanical system possesses only one single bistability.

Figure 3(b–d) display the normalized power forward transmission *T*_*F*_ and backward reflection *T*_*B*_ vary with the detuning Ω when the mode coupling rate *J* is not equal to zero. Specifically, in Fig. 3(b) the mode coupling rate *J* is not big enough, i.e., *J* = 0.2 Ω_*m*_, so the Fano resonance has not yet emerged in this case. While when *J* = 0.4 Ω_*m*_ in Fig. 3(c) and *J* = 0.6 Ω_*m*_ in Fig. 3(d), the system displays rich line-shape structures. A typical asymmetric Fano line-shapes can be generated efficiently near Ω = Ω_*m*_. Physically, the underlying mechanism for generating such Fano resonances is the destructive interference between the forth and back reflections of the optical field through different pathways due to the fact that the WGM microresonator introduces the backward propagating lights via the mode coupling term, i.e., 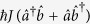 in Eq. (1). By comparing Fig. 3(c) and (d) where the mode coupling rates *J* respectively are 0.4 Ω_*m*_ and 0.6 Ω_*m*_, it is found that the Fano line-shapes change distinctly with the increase of the mode coupling rate *J*. In particular, when we increase the mode coupling rate *J* to a large value of 0.6 Ω_*m*_, the dip of the Fano resonance in the normalized power forward transmission *T*_*F*_ is decreased considerably. We can observe an enhanced Fano line-shape with a resonance maximum (peak) at Ω = Ω_*m*_ and a resonance minimum (dip) at Ω = 1.013 Ω_*m*_. The spectral width between the peak and the dip of the Fano is ΔΩ = 0.013 Ω_*m*_. In our Fano optomechanical system, the forward transmission contrast of the Fano response is as high as approximately 54% [see Fig. 3(d)], which is sufficient for any telecom system^70^. Correspondingly, the peak of the Fano resonance in the normalized power backward reflection *T*_*B*_ is increased due to the energy conservation. At the same time, the spectra of the normalized power forward transmission *T*_*F*_ and backward reflection *T*_*B*_ expand outwards the resonance peak. Hence, we are able to effectively control and tune the Fano line-shapes by appropriately adjusting the coupling rate *J* between the two counterpropagating modes. As shown in ref. 71, the effective mode coupling in the WGM microresonator optomechanical system is usually introduced by internal defect centers or surface roughness. Thus these factors (i.e., manipulating the internal defect centers or surface roughness experimentally) may be used to control and tune the coupling rate *J* between the two counterpropagating modes. In view of rapid advances in micro-nano manufacture technology, we believe that quantitative control of *J* will be accessible in experiments in the near future.

Next, we demonstrate that the line-shapes of the Fano resonance can also be manipulated by varying the power of the control field *P*_*c*_. Figure 4 shows the normalized power forward transmission *T*_*F*_ and backward reflection *T*_*B*_ as a function of the detuning Ω for the four different values of the power of the control field *P*_*c*_. In order to illustrate the dependence of the Fano line-shapes on the power of the control field *P*_*c*_, we keep these parameters *J* = 0.6 Ω_*m*_, *ε*_*p*_/*ε*_*c*_ = 0.05, and *η*_*c*_ = 0.5 fixed, and the other system parameters are exactly the same as those in Fig. 2. In Fig. 4(a), we plot the spectra of the normalized power forward transmission *T*_*F*_ and backward reflection *T*_*B*_ for the case that a control filed of *P*_*c*_ = 10 *μ*W is applied. It can be seen from Fig. 4(a) that one can observe an asymmetric Fano-shape OMIT resonance after zooming the figure but the Fano phenomenon is very weak because the power of the control field is quite small. For the case of *P*_*c*_ = 100 *μ*W in Fig. 4(b), on closer inspection, one can see that a very weak Fano resonance around Ω = Ω_*m*_ starts to appear in the generated transmission and reflection spectra. Interestingly, when the power of the control field is further increased to the values of *P*_*c*_ = 1 mW and 10 mW, we find a pronounced sharp asymmetrical dip (peak) in the transmission (reflection) spectrum, which we identify as the Fano resonance. The Fano resonance becomes more and more stronger, as can be verified from Fig. 4(c,d). From these figures, it is evident that the sharp asymmetric line-shape of the Fano resonance can be formed under the stronger powers of the control field. Alternatively, in Fig. 4 it is also shown that the spectral profiles of the normalized power forward transmission *T*_*F*_ and backward reflection *T*_*B*_ cannot expand outwards the resonance peak, only the heights from the peak-to-dip of the Fano resonance increase gradually, as compared to the results in Fig. 3.

Finally, we turn to discuss how the line-shapes of the Fano resonance can be controlled by the outgoing coupling coefficient *η*_*c*_ and the optomechanical coupling strength *G* in the presence of the mode coupling. In Fig. 5, we first show that the Fano line-shape can be tuned by properly changing the outgoing coupling coefficient *η*_*c*_ under three cavity loading conditions [under (*κ*_*ex*_ < *κ*_*i*_ or *η*_*c*_ < 0.5), critical (*κ*_*ex*_ = *κ*_*i*_ or *η*_*c*_ = 0.5), and over (*κ*_*ex*_ > *κ*_*i*_ or *η*_*c*_ > 0.5) coupling conditions]. Figure 5(a) plots the normalized power forward transmission spectrum *T*_*F*_ as a function of the detuning Ω for the three different values of the outgoing coupling coefficient *η*_*c*_. Specifically, for the case of the outgoing coupling coefficient *η*_*c*_ = 0.1 in Fig. 5(a), the normalized power forward transmission spectrum of *T*_*F*_ is a symmetric W-type of double Lorentzian-like line-shape. As the outgoing coupling coefficient *η*_*c*_ is increased, for example, *η*_*c*_ = 0.5 and 0.8, the asymmetric Fano resonance becomes increasingly obvious. For the sake of clarity, the inset in Fig. 5(a) shows a magnified view of Fano line-shapes near Ω = Ω_*m*_ in a smaller region. Likewise, Fig. 5(b) presents the normalized power backward reflection spectrum *T*_*B*_ varies with the detuning Ω for the three different values of the outgoing coupling coefficient *η*_*c*_. Compared with Fig. 5(a), we can find that the pattern of the Fano resonance is inverted and the sharp peaks appear on the right side of the resonance dip. This is due to different phase shifts between the two resonance modes in the WGM microresonator. Such a situation also occurs in Figs 3 and 4. Lastly, Fig. 6 shows the tunable line-shapes of the Fano resonance by varying the optomechanical coupling strength *G*. In Fig. 6(a) and(b), we can observe the variation of the peak-to-dip spectral spacing by adjusting the optomechanical coupling strength *G*. When the absolute value of coupling coefficient *G* increases, the peak-to-dip spectral spacing increases gradually.

Overall, in view of these detailed discussions above, we can reach the conclusion that the coherent coupling of the two counterpropagating modes, which is neglected in the previous studies^11^,^27^, plays a key role in the generation of asymmetric Fano resonance line-shape. Here the on-chip WGM microresonator optomechanical system provides an easy and robust way to tune and control Fano resonance spectrum by simply changing the experimentally achievable parameters, such as the mode coupling rate *J*, the power of the control field *P*_*c*_, the outgoing coupling coefficient *η*_*c*_, and the optomechanical coupling strength *G*. All of these system parameters are simple and flexible to implement our proposed arrangement. This Fano resonance control will be useful for enhancing the sensitivity of the sensors and designing low-power all-optical switches^54^.

## Discussion

We have proposed a fully on-chip scheme for generating and controlling optical multistability and sharp asymmetric Fano resonance OMIT line-shapes in a three-mode microresonator optomechanical system. The WGM microresonator is driven by an external two-tone laser field consisting of a strong control field and a relatively weak probe field via a tapered fiber. In our model, we consider the two stationary modes cannot be resolved and hence both of them are populated when *J* ≤ *κ* or we assume the condition of *J* > *κ* but not 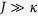 (i.e., the transition coupling region *κ* < *J* < 3*κ*), which are quite different from the previous approach in ref. 27. There are two main results in our study. First, by solving the coupled Heisenberg-Langevin equations and analyzing the stationary state solution, we find that the coupling rate of the two counterpropagating modes, that is parameterized by *J*, plays a key role in manipulating optical multistable properties for the displacement of the mechanical motion. When *J* is very small, there has only one bistable region. Importantly, the bistability can turn into the multistability in the beginning and then becomes the two separated bistable regions with increasing *J*. Second, by using the standard input-output relation and the perturbation method, we analyze in detail the normalized power transmission and reflection spectra of the weak probe laser field. With readily accessible system parameters, we observe the sharp asymmetric Fano resonance line-shapes, which originates from the interference between the forth and back reflections of the optical filed in different pathways. In addition, the sharp asymmetric Fano spectral profile can be controlled and tuned by appropriately changing the mode coupling rate between the two counterpropagating modes, the distance between the cavity and the tapered fiber, the power of the control field, and the optomechanical coupling strength between the counterpropagating modes and mechanical mode, respectively. The scheme could be realized with current physical technology^27^ and all of these system parameters can be adjusted readily under realistic experimental conditions. This investigation may provide new insights into the aspects of the interaction between the WGM microresonator and the mechanical motion. Also, our results will be helpful in practical applications, such as all-optical switches, modulators, and high-sensitivity sensors, etc.

## Methods

### Derivation the normalized power forward transmission *T*_*F*_ and backward reflection *T*_*B*_ of the probe field

Transforming the above Hamiltonian [Eq. (1)] into the rotating frame at the frequency *ω*_*c*_ of the control field by means of 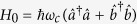, 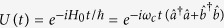, and *H*_*rot*_ = *U*^†^(*t*) (*H* − *H*_0_) *U(t*), we can obtain an effective Hamiltonian as


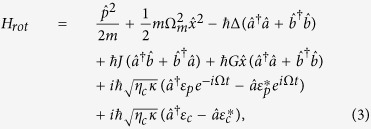


where Δ = *ω*_*c*_ − *ω* and Ω = *ω*_*p*_ − *ω*_*c*_ are the detunings of the control field with frequency *ω*_*c*_ from the cavity field with the frequency *ω* and the probe field with the frequency *ω*_*p*_, respectively.

According to the effective Hamiltonian [Eq. (3)], reducing the operators to their mean values and dropping the quantum and thermal noise terms (all the noise operators have zero mean values), we can get the Heisenberg-Langevin equations of motion as follows:


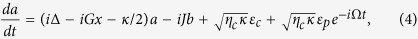



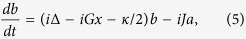



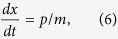



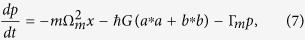


where the decay rates of the cavity field (*κ*) and mechanical oscillator (Γ_*m*_) have been introduced classically.

For the case that the control field is much stronger than the probe field, the perturbation method can be used ref. 27. The control field provides a steady-state solution (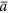, 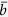, and 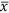) of the system, while the weak probe field is tread as the noise. In this case, the intracavity field and the mechanical displacement can be written as 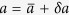, 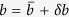, and 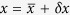. Ignoring the perturbation terms, the steady-state solutions of Eqs (4, 5, 6, 7) can be achieved as


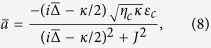



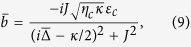



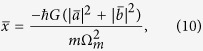


where 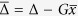. By considering the perturbation for the weak probe field and substituting 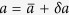, 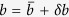, and 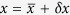 into Eqs (4, 5, 6, 7), we have the results


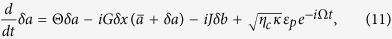



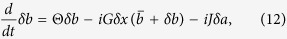



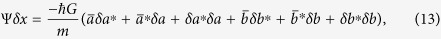


where 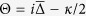 and 
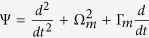
, respectively.

In order to further solve this set of the coupled equations (11, 12, 13), following the method of ref. 27, we introduce the following ansatz for the fluctuation parts of the intracavity field and the displacement of the mechanical mode:


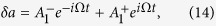



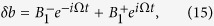



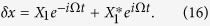


Upon substituting Eqs (14, 15, 16) into Eqs (11, 12, 13) and sorting them by the rotation term *e*^±*i*Ω*t*^, this yields the following five algebra equations:


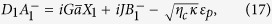



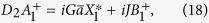



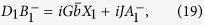



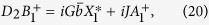



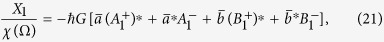


where *D*_1_ = Θ + *i*Ω, *D*_2_ = Θ − *i*Ω, and 
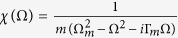
, respectively. It is worth noticing here that the steady-state values 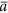 and 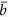 are governed by Eqs (8, 9, 10).

From the above Eqs (17, 18, 19, 20, 21), after tedious but straightforward calculations, the solutions for *X*_1_, 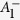, and 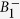 can be derived explicitly as


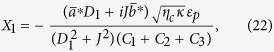



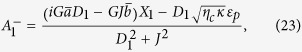



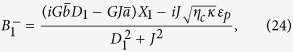


with


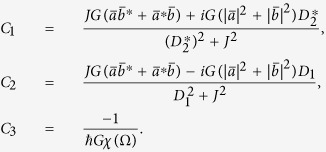


By applying the standard input-output theory^72^,^73^, 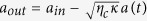 and 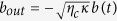, we can arrive at the forward and backward direction output fields as follows:


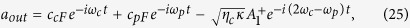



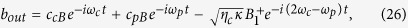


where the three complex coefficients 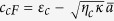, 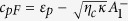, and 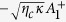 
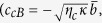

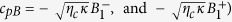
 describe the optical responses at the control-field frequency *ω*_*c*_, the probe-field frequency *ω*_*p*_, and the new frequency 2*ω*_*c*_ − *ω*_*p*_ for the forward (backward) direction output field, respectively. That is to say, from the physical point of view, the expressions (25) and (26) reveal that the forward and backward direction output fields contains two input frequency components (the control field with *ω*_*c*_ and the probe field with *ω*_*p*_) and one additional frequency component (also called Stokes field^11^) with 2*ω*_*c*_ − *ω*_*p*_. In the following, we only focus on the output component at the frequency of the weak probe field like in ref. 27. One also can study the features of the output fields at the frequencies of the control and Stokes fields in an analog way, while the corresponding results are not shown here due to the space limitation.

Hence, the normalized power forward transmission *T*_*F*_ and backward reflection *T*_*B*_ of the probe field can be expressed as


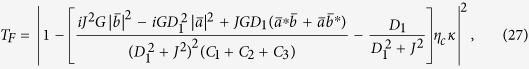



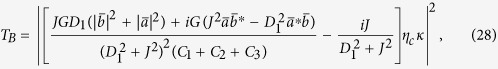


where we have defined *T*_*F*_ = |*c*_*pF*_/*ε*_*p*_|^2^ and *T*_*B*_ = |*c*_*pB*_/*ε*_*p*_|^2^ for convenience, respectively. Equations (27) and (28) are the central results of this paper.

## Additional Information

**How to cite this article**: Zhang, S. *et al*. Optical multistability and Fano line-shape control via mode coupling in whispering-gallery-mode microresonator optomechanics. *Sci. Rep.* **7**, 39781; doi: 10.1038/srep39781 (2017).

**Publisher's note:** Springer Nature remains neutral with regard to jurisdictional claims in published maps and institutional affiliations.
